# Readiness to reduce primary care-associated carbon emissions in England: a cross-sectional survey of clinical and non-clinical staff views

**DOI:** 10.1136/bmjopen-2024-095457

**Published:** 2025-07-18

**Authors:** Olivia Geddes, Helen Twohig, Frederik Dahlmann, Abigail Eccles, Florence Karaba, Ana Raquel Nunes, Rachel Spencer, Jeremy Dale

**Affiliations:** 1 Division of Health Sciences, Warwick Medical School, Coventry, UK; 2 School of Medicine, Keele University, Keele, UK; 3 Strategy & International Business Group, Warwick Business School, Coventry, UK; 4 Nuffield Department of Primary Care, Oxford University, Oxford, UK

**Keywords:** Climate change, health services, general practice, primary care

## Abstract

**Abstract:**

**Objectives:**

To describe current levels of interest and action around decarbonisation in general practice settings, and awareness and use of currently available materials designed to support general practice teams undertake decarbonisation activity.

**Design:**

Cross-sectional, mixed methods, online survey.

**Setting:**

473 general practices in three Integrated Care Board regions in England.

**Participants:**

Multiprofessional general practice staff.

**Results:**

There were 328 responses from 163 (34.5%) practices. Most respondents were general practitioner (GP) partners (98; 29.9%), other clinical staff (93; 28.3%) or managerial staff (76; 23.2%). 229 (69.8%) respondents felt that acting to reduce carbon emissions from primary care is a legitimate part of general practice activity. However, only 44 (13.4%) felt that there is sufficient training and resources to support such activity, and only 59 (18.0%) agreed that there was sufficient leadership from higher levels within the health service to enable this. 58 (35.6%) practices had a lead for sustainability, generally managerial staff (22; 37.9%) or GP partners (17; 29.3%). Compared to other practices, those with a decarbonisation lead reported increased levels of decarbonisation actions currently being undertaken (mean = 5.2 vs 3.1; t(161) = 7.7, p<0.001); use of resource materials that support decarbonisation (79.3% vs 32.3%; χ^2^=31.9, p<0.001); and planned future decarbonisation actions (63.8% vs 15.2%; χ^2^=32.3, p<0.001). Respondents from 47 (28.8%) practices provided free-text comment, mostly noting conflicting workload priorities, limited funding, lack of leadership and practice staff engagement as barriers to decarbonisation activity in their practice.

**Conclusions:**

This survey provides insight into how English general practices and their staff regard decarbonisation activities. The findings highlight the importance of leadership, resources and incentives in driving such activities and have implications for initiatives to help achieve wider decarbonisation goals in healthcare.

**STRENGTHS AND LIMITATIONS OF THIS STUDY**

- Survey responses were from a diverse range of practices from three contrasting Integrated Care Board regions in England.
- Free-text comments permitted further insights into respondents’ views around decarbonisation in general practice.
- The survey received a moderate response rate (34.5%), and it is likely that responding practices are more interested in and actively engaged with decarbonisation efforts.
- The analytic approach used to achieve a practice-level response where more than one member of their team responded revealed a lack of shared knowledge about decarbonisation activity in some practices; this may mean that practices with only one respondent may have under-reported activity that is taking place or being planned.
- The survey was unable to assess the acceptability and extent of the decarbonisation activities reported as having occurred within practices, and the subsequent impact this had on practice decarbonisation.

## Background

The need for urgent action to reduce greenhouse gas emissions to limit the environmental and public health effects associated with climate change is well recognised.^1^,^3^ The effects of climate change are forecast to impact lives both directly and indirectly, exacerbating the social and environmental determinants of health.^4^,^6^ Extreme temperature, an increased frequency of extreme weather events, changing patterns in infectious disease and poor nutrition because of food insecurity will have a negative impact on physical and mental health.^1^ This will have direct consequences for health services globally.^5 7 8^

Not only is healthcare impacted by the changing climate, but it contributes to the problem.^9 10^ Healthcare has a significant carbon emissions footprint; in the UK, the National Health Service (NHS) contributes around 4–5% of total emissions.^11 12^ In 2020, the NHS set a target of achieving net zero emissions by 2040, and since then, healthcare systems in other countries have adopted similar net zero goals.^11 13^ The NHS is expected to be working towards this target, and since 2022, Integrated Care Boards (ICBs) that oversee the provision of local health services have been required to produce Green Plans that describe their strategy for achieving decarbonisation.^11^

It has been calculated that primary care is responsible for 23% of all NHS emissions, derived from the direct delivery of care (eg, building energy, metered dose inhalers), supply chain (including pharmaceuticals and the procurement of medical and non-medical equipment), and staff and patient travel.^9^ There is increasing awareness of the importance of action by general practice to address climate change,^11 14^ and in 2023, the Royal College of General Practitioners (RCGP) included ‘responding to the climate emergency’ as one of their four strategic priorities.^15^

General practice teams are well placed to enact systemic and individual change through their connection with the communities they serve and their provision of comprehensive, person-centred care.^16 17^ General practice staff can act as role models and educators, with the potential to lead and implement actions to tackle climate change while highlighting associated health benefits.^16^,^18^

A recent systematic review identified numerous actions that can be taken in general practice to support decarbonisation.^19 20^ Studies from the USA, Europe and Australia have explored ways of reducing carbon emissions through interventions aimed at energy and waste reduction,^21 22^ supply chains and procurement,^22^ cervical screening approaches,^23^ asthma inhaler prescribing,^24^ climate-sensitive health counselling,^25 26^ patient and staff travel^27 28^ and social and nature prescribing.^29^,^32^

Although national guidance is lacking on how general practice can achieve net zero,^33^ there are a growing number of resource materials available to support practices undertaking decarbonisation actions (table 1). However, little is understood about how widely such resources are being used in general practice, and their overall impact.

**Table 1:** Examples of resources available to support decarbonisation initiatives in National Health Service general practice

Resource	Description
Green Impact for Health Toolkit(https://greenimpact.nus.org.uk/green-impact-for-health/)	A free sustainability-related quality improvement (SusQI) scheme listing over 100 actions that can improve the environmental sustainability and quality of any general practice.^51^
Greener Practice Network(https://www.greenerpractice.co.uk/#:~:text=Greener%20practice%20can%20improve%20health%20now,%20mitigate%20the%20climate%20crisis)	A network of general practice staff to share resource materials and learning around decarbonising general practice.^38^
High Quality and Low-Carbon Asthma Care Toolkit(https://www.greenerpractice.co.uk/high-quality-and-low-carbon-asthma-care/#:~:text=This%20toolkit%20is%20designed%20to%20help%20UK%20general%20practices%20improve)	A toolkit designed to help general practices improve asthma outcomes while also reducing carbon emissions through step-by-step quality improvement projects.^52^
General Practice Non-clinical Carbon Calculator(https://www.gpcarbon.org/#/)	A tool designed to calculate a carbon equivalent footprint of a general practice.^53^
RCGP Net-zero e-Learning Hub(https://elearning.rcgp.org.uk/course/view.php?id=650&dm_t=0,0,0,0,0#:~:text=The%20RCGP%20net%20zero%20hub%20contains%20eLearning%20modules%20written%20by)	A platform containing e-learning modules relating to sustainability in general practice.^54^

To start to address this, we undertook a general practice staff survey with the aim of establishing current levels of general practice staff interest in and engagement with decarbonisation initiatives, including their awareness and use of relevant, widely accessible resource materials.

## Methods

The methods and results are reported in conjunction with the Checklist for Reporting Results of Internet E-Surveys (online supplemental file 1).^34^ An open questionnaire was developed using the online survey platform Qualtrics XM, Provo (V.7.24) to collect data from a convenience sample of clinical, managerial and administrative staff working in general practice in three contrasting ICB regions of England. These areas were chosen to reflect a range of rural and urban settings, levels of deprivation, ethnic diversity and known activity in relation to decarbonisation.

The survey opened with an introductory page, explaining the survey’s purpose, duration, incentives, details around data storage and contact information for the study’s principal investigator. It had 10 items across 5 pages, or 8 items across 3 pages with the use of adaptive questioning to ensure relevance to the respondent depending on their previous responses. All survey items, except the final free-text question, were mandatory, and response validation was used on all questions, where appropriate. The survey design was informed by a systematic review,^19 20^ and the Normalisation Measure Development questionnaire evaluation tool, a sociologically informed, 23-item instrument for measuring implementation processes from the perspective of professionals directly involved in the work of implementing complex interventions.^35^ The survey design received additional input from the study’s stakeholder advisory group, including representatives from national policy maker and third sector organisations involved in supporting decarbonisation in general practice, such as Greener NHS^36^ and SEE Sustainability.^37^

The length of the survey was limited, allowing it to be completed in under 5 min to encourage uptake. To prevent duplicate entries, participants were only allowed to visit the survey once from the same internet protocol address and had 7 days to complete the survey once started. Respondents were able to review and change their answers prior to submission using a back button. Survey questions and functionality were refined through piloting with general practice staff outside of the recruitment areas, including practice managers, general practitioners (GP) and GP trainees (online supplemental file 2).

### Participant recruitment

Eligible participants included GP partners and their employees from practices in Coventry and Warwickshire, Birmingham and Solihull, and South Yorkshire ICBs. Within these regions, there were a total of 473 practices with a registered population of over 4.2 million patients.

A multistrand approach was taken to raising awareness of the survey. This included disseminating information about the survey (including a Quick Response (QR) code and weblink to the survey webpage) at regional level and Primary Care Network (PCN) level through newsletters and educational events (online supplemental file 3). In addition, local general practice federations and relevant organisations, such as the Greener Practice network,^38^ Centre for Sustainable Healthcare^39^ and Greener NHS,^36^ were contacted to publicise the survey. This was supplemented by the work of local National Institute for Health Research Clinical Research Network representatives who disseminated the survey to research active practices in each area.

The survey was open to responses between 16 November 2023 and 16 February 2024. To incentivise participation, a tree was planted through NHS Forest^40^ on behalf of each practice that had at least one respondent. Respondents could also opt into receiving a certificate of participation, and for entry into a prize draw to win one of five £50 shopping vouchers.

### Patient and public involvement

Patients were not directly involved in the design, conduct or reporting of the study due to its focus on general practice staff. However, patient and public representatives were involved in the conceptualisation of the wider GPNET-0 (General Practice Net Zero) Study (www.warwick.ac.uk/gpnet0) of which this survey is part.^41^

### Data analysis

Data from complete questionnaires were downloaded from the survey platform into Microsoft Excel (Microsoft Corp, Redmond, Washington, USA) and stored in password-protected files and devices, on secure University of Warwick servers. Numerical data were analysed using statistical methods supported by Qualtrics’s analytic software (V.7.24), and IBM SPSS (V.29.0.1.0 (171)). Survey data were anonymised ahead of analysis. Job roles of respondents were categorised into four main groups (managerial (eg, practice managers, business managers), GP partners, clinical (eg, salaried GPs, practice nurses) and non-clinical (eg, receptionists, administrative staff)). Index of multiple deprivation (IMD) score and practice list size were grouped by quartile.

The primary analysis was at practice level to ensure the data was not weighted towards practices who had submitted multiple responses. Analysis of general practice staff views around decarbonisation in general practice was at an individual level. To produce a single response for each practice where there were multiple respondents, we took an amalgamative approach, prioritising the most active response received. For example, if at least one of multiple respondents noted that their practice was considering future decarbonisation actions, their answer would be selected for the practice response.

Descriptive statistics including absolute and relative frequencies were used to describe responses. T-tests (one-sample and independent) were performed to compare sample means. χ^2^ and Fisher exact tests were used to test for independence between categorical variables. Means, medians, IQR and test statistics were reported as appropriate.

Free-text responses were thematically analysed using Lumivero NVivo (V.1.7.1 (1534)). Themes were inductively identified from the data.^42^

## Results

### Response rate

There were 328 responses from a total of 163/473 (34.5%) eligible practices; 54 (33.1%) practices had more than one respondent. The practice response rate was similar across the three ICB regions.

The characteristics of practices are shown in table 2. Overall in terms of list size, participating practices were representative of English practices (sample mean: 10 404, national mean: 9803, t(156)=1.428, p=0.155), but had higher levels of deprivation (sample mean: 27.8, national mean: 21.7, t(162)=5.667, p<0.001) as measured by the IMD score at practice level (2019).

**Table 2:** Characteristics of responding practices

	Total sample	Birmingham and Solihull	Coventry and Warwickshire	South Yorkshire
Number of responding practices, n (%)	163	54 (33.1)	60 (36.8)	49 (30.1)
Average practice list sizeRange (rounded)	10 4041400–30 000	93601500–30 000	10 8522300–27 700	99111400–20 700
Average deprivation scoreRange*	27.85.2–54.4	35.55.7–53.7	20.07.3–39.6	28.75.2–54.4
Rural (%)Urban (%)†	15 (9.2)148 (90.8)	1 (1.9)53 (98.1)	10 (16.7)50 (83.3)	4 (8.2)45 (91.8)

* Measured by the index of multiple deprivation score at practice level (2019).

† Measured by the rural urban classification at practice level (2011).

Respondents included GP partners (98; 29.9%), clinical staff (93; 28.3%), managerial staff (76; 23.2%) and non-clinical staff (61; 18.6%). Clinical staff included salaried GPs, GP trainees, practice nurses, pharmacists, healthcare assistants, physician associates and other direct-patient care roles. Managerial staff were mainly practice managers, and non-clinical staff were mainly receptionists and secretaries.

### Decarbonisation lead at practice level

Respondents from 58 (35.6%) practices reported that they had a practice lead for sustainability/decarbonisation. However, in 19 (32.7%) of them, 1 or more respondents were unaware that their practice had a lead. The identified leads were managerial staff (22 practices, 37.9%), GP partners (17 practices; 29.3%) and salaried GPs (8 practices; 13.8%). In six practices with a lead, there was inconsistency in who this was, with respondents reporting different individuals as holding this position.

### Decarbonisation activity at practice level

**Figure 1:**
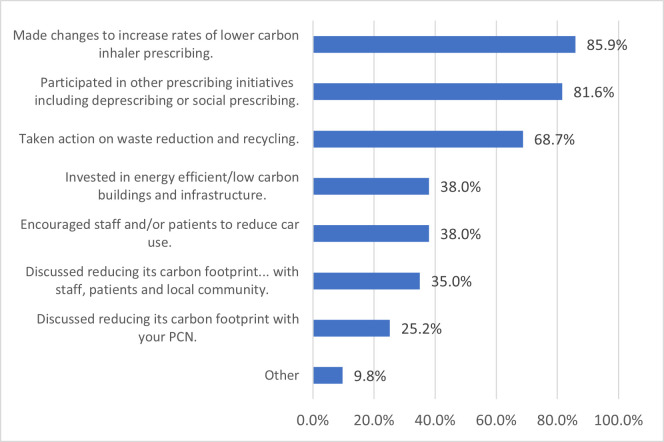
Practice-reported activity to support decarbonisation (n=163). PCN, Primary Care Network.

Respondents were presented with a list of decarbonisation actions and asked to identify any that had been undertaken in their practice in the past year (figure 1). Those most frequently reported related to inhaler prescribing (140; 85.9%), other prescribing initiatives (133; 81.6%), and waste reduction and recycling (112; 68.7%). Nine practices (5.5%) reported no decarbonisation actions to have taken place.

The median number of reported actions per practice was 4 (IQR 2–5). Practices with a decarbonisation lead reported more actions being undertaken compared with those with no identified lead (means=5.2 and 3.1, respectively; t(161)= 7.7, p<0.001).

For the 16 (9.8%) practices where a respondent had selected ‘other’, actions relating to energy (9; 56.3%) and waste reduction and recycling (4; 25.0%) were noted in the free-text box.

**Figure 2:**
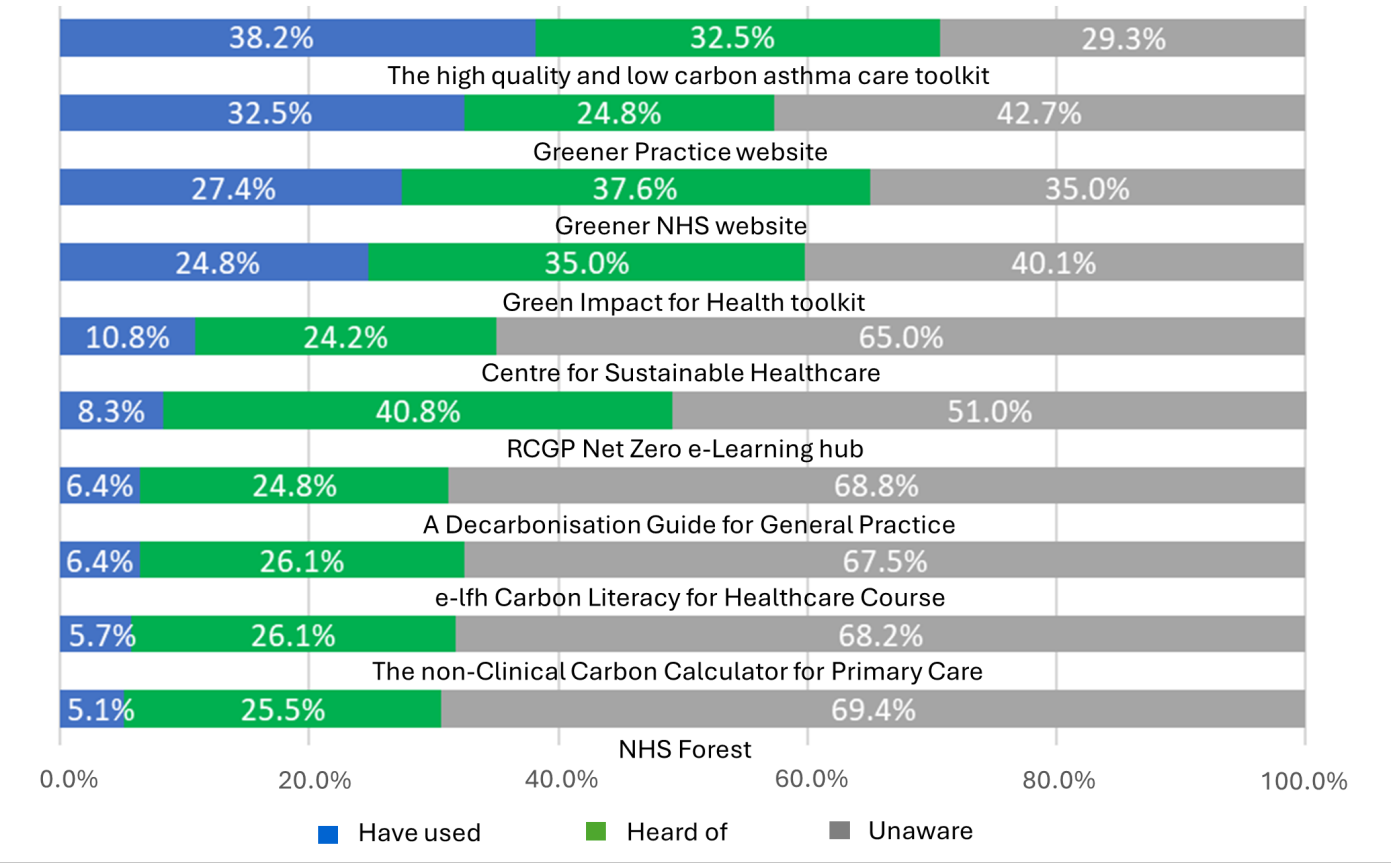
Practice-level awareness and use of resources designed to support decarbonisation in general practice (n=157).

Respondents were asked to indicate awareness and use of a range of currently available resource materials designed to support general practice teams plan and undertake decarbonisation activities (figure 2). For 20 (12.7%) practices, respondents were completely unaware of these resources, and a further 57 (36.3%) were aware of one or more but had not used any. Two out of the four most used resources were practical toolkits, the most frequently used resource being the ‘High quality and low carbon asthma care toolkit’; this was reported as having been used by 60 (38.2%) practices, a finding that aligned with inhaler prescribing having been reported as the most commonly targeted area for decarbonisation.

47 (28.8%) practices were considering undertaking additional decarbonisation actions: 25 (53.2%) of these related to energy usage, 12 (25.5%) to reducing paper usage and 8 (17.0%) to inhaler prescribing and disposal.

Presence of a practice decarbonisation lead was associated with a practice having used at least one of the listed resource materials (46 (79.3%) vs 34 (32.3%) for practices that lacked an identified lead; χ^2^=31.9, p<0.001). Presence of a decarbonisation lead was also associated with future actions being planned in a practice (31 (63.8%) compared with 16 (15.2%) for practices without an identified lead; χ^2^=32.3, p<0.001).

### Views about the importance of decarbonisation in general practice

Respondents’ level of agreement with statements about decarbonisation in general practice is shown in table 3.

**Table 3:** Respondent views about the importance of decarbonisation in general practice (n=328)

	Strongly agree	Agree	Neither agree nor disagree	Disagree	Strongly disagree	Don’t know
I believe that acting to reduce greenhouse gas emissions from primary care is a legitimate part of my role (n (%)).	86 (26.2)	143 (43.6)	71 (21.6)	10 (3.0).	5 (1.5)	13 (4.0)
We have sufficient training and resources to implement decarbonisation actions (n (%)).	5 (1.5)	39 (11.9)	89 (27.1)	107 (32.6)	47 (14.3)	41 (12.5)
We have management support from the ICB and/or PCN to take actions in this area (n (%)).	7 (2.1)	52 (15.9)	81 (24.7)	65 (19.8)	40 (12.2)	83 (25.3)
Actions to decarbonise general practice disrupt our normal way of working (n (%)).	10 (3.0)	45 (13.7)	98 (29.9)	122 (37.2)	26 (7.9)	27 (8.2)
There are key people in our team who work to drive the decarbonisation agenda forwards and get others involved (n (%)).	41 (12.5)	95 (29.0)	81 (24.7)	55 (16.8)	18 (5.5)	38 (11.6)
Staff in our PCN have environmental sustainability as a shared value (n (%)).	20 (6.1)	102 (31.1)	108 (32.9)	28 (8.5)	6 (1.8)	64 (19.5)
Staff in our general practice have environmental sustainability as a shared value (n (%)).	27 (8.2)	130 (39.6)	105 (32.0)	36 (11.0)	4 (1.2)	26 (7.9)

ICB, Integrated Care Board; PCN, Primary Care Network.

The majority agreed that acting to reduce carbon emissions from primary care was a legitimate part of their role (229; 69.8%). However, staff working in non-clinical roles were less likely to agree with this statement, with only 24 (39.3%) members of non-clinical staff agreeing, compared with 77 (82.8%) respondents in clinical roles (χ^2^=38.1, p<0.001). Respondents who reported their practice as having a lead for decarbonisation were more likely to agree with this statement (88 (82.2%) vs 141 (63.8%); χ^2^=11.6, p<0.001). These respondents were also more likely to agree that they had decarbonisation as a shared practice value (69 (64.5%) vs 88 (39.8%); χ^2^=17.6, p<0.001).

Few (44; 13.4%) respondents agreed with the statement that they had sufficient training and resources to be able to implement decarbonisation actions. Notably, only four (4.1%) GP partners agreed, significantly less than for those in other job roles (p=0.003).

In addition, few (59; 18.0%) respondents reported they had adequate support from the ICB and/or PCN to take actions relating to decarbonisation. GP partners were also less likely to agree with this statement, with only seven (7.1%) individuals agreeing (χ^2^=12.4, p=0.006).

There were mixed views over the extent to which leadership within their general practice team was driving decarbonisation actions, and about environmental sustainability as a shared value at PCN and general practice levels (see table 3).

### Analysis of free-text comments

In total, 62 (19.0%) respondents from 47 (28.8%) practices provided a free-text response at the end of the survey. GP partners made up the highest proportion of these (28; 45.2%), followed by clinical staff (22; 35.5%). Barriers to action relating to workload, lack of time, and funding were prominent themes. Lack of support for practice-level leadership and challenges engaging the whole team were also mentioned, in addition to inadequate higher-level NHS support for decarbonisation actions. While most comments reflected a willingness to undertake decarbonisation actions, the barriers were often experienced as too overwhelming to allow this within their practice. Table 4 provides quotes illustrative of the themes identified.

**Table 4:** Free-text quotes illustrating identified themes relating to the implementation of decarbonisation actions in general practice

Themes	Illustrative quotes (with respondent codes: Rnumber is the respondent number, and each letter signifies a job role (M, managerial staff; GPp, GP partner; C, clinical staff; NC, non-clinical staff))
Practice workload	“In my practice the doctors are engaged but too overwhelmed with work to take an active role.”—R42, C“It’s looked upon as extra on top of an already impossible workload.”—R40, C
Limited time and capacity	“Honestly although I think this is important I just think with everything else we have to get through on a daily basis there is no headspace or time for anything else!”—R10, GPp“I have no specific allocated time to do anything”—R40, C“General practice is on its knees and this impacts our energy to do anything that takes even 1 second more. Implementing greener choices takes time and resources that we do not have.”—R7, GPp
Limited funding	“We do not have sufficient resources or funding to push forward our decarbonisation plans.”—R54, C“When even simple initiatives- like paper recycling- will cost us several hundred pounds a year when budgets are so tight.”—R44, GPp
Higher-level support	“Does not seem to be a priority for PCNs, Federation or ICB.”—R36, C“I am interested but feel we need more support from our ICB.”—R12, GPp
Leadership	“There is no-one with a set role on this.”—R48, GPp“General practice needs… passionate leaders to drive forwards decarbonisation.”—R49, GPp“Despite all my enthusiasm [as someone leading decarbonisation actions], I am not paid and the role is not official, I’ve just developed a name for being ‘green’.”—R40, C
Engaging the wider general practice workforce	“Involving all staff groups especially reception is a challenge.”—R32, GPp“…little enthusiasm from the rest of the workforce, in fact antipathy has become overwhelming.”—R40, C

## Discussion

### Principal findings

This survey has identified a high level of acceptance that acting to reduce carbon emissions from primary care is a legitimate concern of general practice. Several issues affecting how general practice teams are engaging with decarbonisation initiatives appear to be independent of practice area, setting or size. It was widely felt that there is insufficient training and resources, or guidance and support from higher levels of the NHS, to enable practice-level activity.

Just over a third of general practices reported that they had a lead for decarbonisation/sustainability, most frequently a practice manager or a GP partner. Practices with a designated lead reported a considerably higher number of decarbonisation actions being undertaken compared with those without a lead. They were much more likely to be aware of and using resources designed to support decarbonisation and were much more likely to be proactively planning future actions. This proactive approach suggests that the presence of a lead not only encourages action but also fosters a forward-thinking approach to sustainability within the practice. However, there were some inconsistencies within the same practices when identifying which team member was the decarbonisation lead, suggesting a lack of clarity, lack of awareness or poor communication.

When invited to expand on their answers, free-text comments re-iterated leadership as an important facilitator to decarbonisation, both at practice level and from higher levels of the NHS. However, respondents reflected on the informal, voluntary nature of many of these leadership roles at practice level, undertaken by enthusiastic staff members who lack protected time to plan and implement activities, leading to questions over the long-term sustainability of these positions. Non-clinical staff members seemed less engaged with decarbonisation in their practice; they were less likely to feel that acting to reduce greenhouse gas emissions was a legitimate part of their role. This was reflected in the free-text comments, with engaging the wider practice workforce identified as a barrier to decarbonisation. Other barriers identified were around practice capacity, including workload, time and a lack of funding, in addition to a lack of higher-level (ICB, PCN) support for decarbonisation.

### Strengths and limitations

To our knowledge, this is the first survey to assess general practices’ engagement with decarbonisation action in England. A key strength is that it was undertaken in three contrasting regions that covered a range of urban and rural environments and varying levels of deprivation. Despite a broad range of incentives to completing the survey and promoting the survey through multiple channels, the survey only achieved a moderate practice response rate (34.5%). It is probable that those who responded are more engaged with decarbonisation efforts and place greater importance on general practice actively taking actions to accelerate this than those who did not. Hence, it is important that the findings, particularly those relating to the extent to which activities are currently occurring, are considered within this context.

A strength was that we did not limit the number of staff members from each practice that could participate in the survey as we wanted to capture diverse perspectives on decarbonisation plans and activities. This approach allowed us to see that, within practices that had several respondents, there were significant differences in opinions and varying levels of awareness about their approach to decarbonisation.

To generate a single practice-level response for questions about specific decarbonisation actions, either taken or planned, we assumed that if at least one respondent from that practice reported a positive answer, then this was a correct indicator, in contrast to any negative responses from other staff members at the same practice which were attributed to lack of awareness. While this pragmatic, amalgamative approach helped address inconsistent internal knowledge, it may have led to some inaccuracies. This is especially likely in practices with only one respondent, particularly if that individual did not hold a leadership or managerial role related to decarbonisation and was therefore less aware about initiatives led by others.

The use of free-text comments, in addition to the survey’s closed questions, added to the understanding of the respondents’ views. However, these data were limited in breadth and depth, indicating a need for more detailed research.

Finally, we were unable to assess the acceptability and extent of the activities reported as having occurred within practices, how resource materials had been used and, most importantly, the measurable impact that this had. It cannot be assumed that all initiated activities were undertaken successfully in terms of achieving their intended purpose. While this was beyond the scope of this survey, this is currently being explored through longitudinal investigation as part of the GPNET-0 Study.^41^

### How this compares to other studies

A recent systematic review demonstrated complexity surrounding the implementation of decarbonisation actions in general practice, identifying four main factors including patient and community engagement, knowledge and awareness, personal and professional integration, and practice management and leadership.^20^ A common finding in this literature was the role of GPs as role models and sources of information around the impact of climate change on human health for patients and communities.^20 43 44^

The current study identified the key role that leadership within practices plays in ensuring engagement with decarbonisation actions. This is supported by international research that has recognised the importance of local, regional and national leadership, and a general practice culture that values environmental sustainability.^21 45^ Barriers that limit or prevent action include lack of time, lack of guidance, workforce shortages, lack of financial incentives, and potential costs,^2143^,^46^ issues that were echoed in the qualitative findings of our survey. Such factors not only affect general practice but also have been shown to apply across other healthcare settings.^47^

### Implications for practice and policy makers

Several key policy recommendations emerge from this study that are relevant to achieving decarbonisation in primary care. These include the importance of encouraging practice-level sustainability leadership and the uptake and use of resource materials available to support decarbonisation activity, addressing financial and workload barriers, targeting non-clinical staff engagement, strengthening higher-level NHS leadership, and incentivising success (box 1). Where action is incentivised, there was evidence that this had proved effective. For example, most practices reported having taken action around inhaler prescribing; this is likely to reflect the Impact and Investment Fund in 2022/2023 which financially incentivised targets to encourage general practices in England to reduce the environmental impact of inhalers.^48^ Presence of a sustainability/decarbonisation lead was identified as a key factor that supported decarbonisation activities. Acting as a decarbonisation lead is likely to take time and effort, and policy makers and commissioners should consider ways of resourcing, incentivising and rewarding such activities. In the context of the workforce, workload challenges and conflicting priorities facing NHS general practice,^49 50^ it is particularly urgent that policy makers and commissioners address these requirements if progress towards achieving the NHS Net Zero targets is to be maintained.

Box 1 **Key implications for policy makers**

**Leadership and resources**

Dedicated leadership for decarbonisation within general practices, combined with adequate resources, is required. Policies should encourage the establishment of decarbonisation/sustainability roles and provide the necessary support and incentives, through funding, training and protected time for undertaking such activity.

**Address financial and workload barriers**

Policy makers should address concerns about the pressure of existing workloads and the lack of funding to support decarbonisation. Financial incentives, such as those successfully used to promote environmentally friendly inhaler prescribing, could be expanded to other areas. Policies are also needed that ring-fence staff time to engage in decarbonisation initiatives.

**Target non-clinical staff engagement**

Non-clinical general practice staff appear less likely to view decarbonisation as part of their role. To achieve comprehensive engagement, targeted education and awareness campaigns should be considered for this group, emphasising the importance of their contribution to the National Health Service (NHS) Net Zero goals.

**Strengthen higher-level NHS leadership, support and guidance**

Integrated Care Boards (ICBs) and Primary Care Networks (PCNs) are currently viewed as providing inadequate leadership and support. There should be greater visibility given to developing and implementing clear guidance and support to practices, in addition to facilitating knowledge sharing between practices.

**Celebrate success and share best practice**

Systems for recognising and rewarding practices for successful decarbonisation efforts should be considered, including sharing best practices and showcasing successful initiatives.

This study was conducted as part of the wider GPNET-0 Study, aiming to explore how institutional, organisational, professional and patient factors influence the implementation and sustainability of actions to mitigate the greenhouse gas emissions associated with general practice^41^ (www.warwick.ac.uk/gpnet0). This study is undertaking an in-depth exploration that builds on the findings of this survey to address key questions relating to the system-wide factors that influence the uptake and implementation of decarbonisation activities in general practice.^34 35^

## Supplementary material

10.1136/bmjopen-2024-095457 online supplemental file 1

10.1136/bmjopen-2024-095457 online supplemental file 2

10.1136/bmjopen-2024-095457 online supplemental file 3

## Data Availability

Data are available upon reasonable request.
