# Supplementary material for: Readiness to reduce primary care-associated carbon emissions in England: a cross-sectional survey of clinical and non-clinical staff views
Source: BMJ Open. 2025 Jul 18;15(7):e095457. doi: 10.1136/bmjopen-2024-095457 (PMC12273146; doi:10.1136/bmjopen-2024-095457)
Supplement: online supplemental file 2 [file bmjopen-15-7-s002.docx]

Supplemental file 2- Electronic survey

Implementation of decarbonisation actions in General Practice to help achieve a net zero NHS (GPNET-0 Study)

**General Practice Survey**

Thank you for taking part in this survey about whether and how general practices are attempting to reduce the greenhouse gas (GHG) emissions associated with their work.

The survey is open to anyone who works in general practice in Coventry and Warwickshire ICB, Birmingham and Solihull ICB and South Yorkshire ICB.

**It takes less than 5 minutes – and once completed, you can enter into a prize draw to win one of five £50 shopping vouchers.**

The survey’s findings will inform the future work of the GPNET-0 Study, an NIHR-funded research project that aims to **understand how general practice is implementing actions to decarbonise** (i.e., reduce its carbon footprint), and to generate recommendations on how to accelerate this process towards achieving a net zero NHS. Information about the GPNET-0 study can be found [here](https://warwick.ac.uk/fac/sci/med/research/hscience/apc/qualityandsafety/gpnet-0/wp7/).

**In addition,** [**NHS Forest**](https://nhsforest.org/) **will plant one tree at a healthcare site for each practice with one or more staff completing the survey. We hope that this will contribute to several hundred trees being planted.**Practices will also receive a **certificate** for the waiting room in recognition of their support for the GPNET-0 study and the tree that has been planted.

**We are keen to hear from you** regardless of whether your general practice is or isn’t attempting to reduce its GHG emissions at present.

**Who is the research team**
 We are a multi-disciplinary team of researchers at Warwick, Keele and Birmingham Universities, led by Dr Raquel Nunes and Professor Jeremy Dale. Full details of the research team can be found [here](https://warwick.ac.uk/fac/sci/med/research/hscience/apc/qualityandsafety/gpnet-0/wp7/). The research is funded by the National Institute for Health and Care Research (NIHR) and has the support of Birmingham and Solihull ICB, Coventry and Warwickshire ICB, and South Yorkshire ICB. The study has received **HRA and Health and Care Research Wales (RCRW) Approval** (REC reference: 23/PR/1169).  
  
 **Taking part in the survey**

By completing the survey, you are consenting for your data to be used in the study. All data will be held securely at the University of Warwick, and no identifiable practice or personal information will be saved with the survey answers. The survey asks for your general practice name and postcode to allow us to describe the practices that respond, and hence the number of trees that will be planted by NHS Forest. All identifiable information (such as email addresses and names provided for entry to the Prize Draw, Certificate of Participation, and Mailing List) will be separated from the rest of the data prior to data analysis and stored separately in order to protect the anonymity of survey responses.

**Page break**

Section 1 Firstly, we would like to ask you some questions about your general practice and your role.

*Q1 Name and postcode of your general practice.

- General practice name

__________________________________________________

- General practice postcode __________________________________________________

*Q2 Which ICS area is your general practice in?

- Birmingham and Solihull
- Coventry and Warwickshire
- South Yorkshire

*Q3 What is your job title?

- GP Partner
- Salaried GP
- GP Trainee
- Practice Nurse
- Advanced Nurse Practitioner
- Physician Associate
- Practice Manager
- Receptionist
- Health Care Assistant
- Pharmacist
- Other (please specify)

__________________________________________________

*Q4 Do you know if your general practice has a lead person for sustainability/ net zero/ decarbonisation?

- Yes
- No
- I don't know

**Page break**

Display This Question:

If Do you know if your general practice has a lead person for sustainability/ net zero/ decarbonisat... = Yes

*Q4.1 What is the job title of the lead person?

- GP Partner
- Salaried GP
- GP Trainee
- Practice Nurse
- Advanced Nurse Practitioner
- Physician Associate
- Practice Manager
- Receptionist
- Health Care Assistant
- Pharmacist
- Other (please specify)

__________________________________________________

**Page break**

Section 2 We would like to know about any activity that your general practice has recently taken to reduce its carbon footprint.

*Q5 In the last year, are you aware of your general practice team taking any actions towards decarbonising its activities? (Tick all that apply)

- Encouraged staff and/or patients to reduce car use.
- Invested in energy efficient/low carbon buildings and infrastructure (e.g., insulation, new glazing, solar panels, heat pump, LED lighting, etc.).
- Made changes to increase rates of lower carbon inhaler prescribing.
- Participated in other prescribing initiatives including deprescribing or social prescribing, even if the primary motivation wasn’t to reduce carbon emissions.
- Taken action on waste reduction and recycling.
- Discussed reducing its carbon footprint through communications and engagement with staff, patients and local community.
- Discussed reducing its carbon footprint with your PCN.
- Other (please specify) __________________________________________________
- I don't know.
- No actions taken in any area.

Skip To: Q6 If In the last year, are you aware of your general practice team taking any actions towards decarbon... = No actions taken in any area.

| 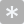 |
| --- |

**Page break**

*Q5.1 Please indicate which of the following resources you have heard of or have used within your general practice? (Please provide an answer against each initiative)

|  | Have used | Heard of | Unaware |
| --- | --- | --- | --- |
| [Greener NHS Website](https://www.england.nhs.uk/greenernhs/wp-content/uploads/sites/51/2022/07/B1728-delivering-a-net-zero-nhs-july-2022.pdf) |  |  |  |
| [NHS Forest](https://nhsforest.org/) |  |  |  |
| [Centre for Sustainable Healthcare website/ courses](https://sustainablehealthcare.org.uk/) |  |  |  |
| [The Green Impact for Health Toolkit](https://greenimpact.nus.org.uk/green-impact-for-health/#:~:text=Green%20Impact%20for%20Health%20is%20part%20of%20the,over%201400%20%2815%25%29%20General%20Practices%20across%20the%20UK.) |  |  |  |
| [Greener Practice Website](https://www.greenerpractice.co.uk/) |  |  |  |
| [The high quality and low carbon asthma toolkit](https://www.greenerpractice.co.uk/high-quality-and-low-carbon-asthma-care/) |  |  |  |
| [RCGP net zero hub e-learning modules](https://www.rcgp.org.uk/Blog/Greener-practice-initiative) |  |  |  |
| [The non-clinical carbon calculator for primary care](https://www.gpcarbon.org/#/) |  |  |  |
| [A decarbonisation guide for general practice](https://elearning.rcgp.org.uk/pluginfile.php/185918/mod_resource/content/4/8514%20-%20RCGP%20-%20Net%20Zero%20-%20Decarbonising%20Guide%20AW2.pdf) |  |  |  |
| [e-lfh carbon literacy for healthcare course](https://www.e-lfh.org.uk/programmes/carbon-literacy-for-healthcare/) |  |  |  |
| Other (please specify) |  |  |  |

**Page break**

*Q6 Are you aware of any new or additional actions aimed at decarbonising its activities that your general practice is currently considering?

- Yes (which?) __________________________________________________
- No
- I don't know

*Q7 We would like to understand your feelings about the importance of decarbonisation activities and the role you feel general practice should take. Please indicate your level of agreement with each of the following statements.

|  | Strongly agree | | Agree | | Neither agree nor disagree | | Disagree | | Strongly disagree | | Don't know |
| --- | --- | --- | --- | --- | --- | --- | --- | --- | --- | --- | --- |
| I believe that acting to reduce greenhouse gas emissions from primary care is a legitimate part of my role. |  |  | |  | |  | |  | |  | |
| Staff in our general practice have environmental sustainability as a shared value. |  |  | |  | |  | |  | |  | |
| Staff in our PCN have environmental sustainability as a shared value. |  |  | |  | |  | |  | |  | |
| There are key people in our team who work to drive the decarbonisation agenda forwards and get others involved. |  |  | |  | |  | |  | |  | |
| Actions to decarbonise general practice disrupt our normal way of working. |  |  | |  | |  | |  | |  | |
| We have management support from the ICB and/or PCN to take actions in this area. |  |  | |  | |  | |  | |  | |
| We have sufficient training and resources to implement decarbonisation actions. |  |  | |  | |  | |  | |  | |

Q8 If you would like to expand on any of your answers or make any comments about this survey, please do so here:

________________________________________________________________

________________________________________________________________

________________________________________________________________

________________________________________________________________

________________________________________________________________

*Q9 Do you think your general practice would be interested in participating in the next stage of the GPNET-0 study as a case study site?

 Information about this opportunity is available here (www.warwick.ac.uk/gpnet0). Your practice, regardless of the extent to which it is currently undertaking actions aimed at decarbonisation, would be facilitated to undertake one or more new actions over a period of 9-12 months. This will be evaluated by the research team. Each case study practice will receive £2500 for participation. If you would like to learn more about this, please indicate below and the research team will contact your practice to discuss this.

- Yes, please can the research team contact my practice to discuss what is involved in being a GPNET-0 case study site.
- No, thank you.

*Q10 Would you like your practice to receive a Certificate of Participation, in recognition of support of the GPNET-0 Study, and the tree that has been planted on behalf of the practice?

- Yes, I would like my practice to be sent a Certificate of Participation by email.
- No, thank you.

*Q11 Would you like to be entered into our prize draw to win one of five £50 shopping vouchers?

- Yes, I would like to be entered into the prize draw for completing the survey.
- No, thank you.

*Q12 Would you like to be added to the GPNET-0 Study's mailing list, to receive bi-monthly newsletters on study progression?

- Yes, I would like to sign up to the GPNET-0 Study's mailing list.
- No, thank you.

**Page break**

Display This Question:

If Do you think your general practice would be interested in participating in the next stage of the... = Yes, please can the research team contact my practice to discuss what is involved in being a GPNET-0 case study site.

Or Would you like your practice to receive a Certificate of Participation, in recognition of support... = Yes, I would like my practice to be sent a Certificate of Participation by email.

*Q13 Please enter contact details for yourself and your practice below. This shall be separated from the rest of the data prior to analysis and used only for contact regarding the incentives you previously opted into.

- Contact name __________________________________________________
- Email address __________________________________________________
- Practice name __________________________________________________
- Practice email address

__________________________________________________

- Practice phone number

__________________________________________________

Display This Question:

If Do you think your general practice would be interested in participating in the next stage of the... = No, thank you.

And Would you like your practice to receive a Certificate of Participation, in recognition of support... = No, thank you.

And If

Would you like to be entered into our prize draw to win one of five £50 shopping vouchers? = Yes, I would like to be entered into the prize draw for completing the survey.

Or Would you like to be added to the GPNET-0 Study's mailing list, to receive bi-monthly newsletters... = Yes, I would like to sign up to the GPNET-0 Study's mailing list.

*Q13 Please enter your contact details below. This shall be separated from the rest of the data prior to analysis and used only for contact regarding the incentives you previously opted into.

- Contact name __________________________________________________
- Email address __________________________________________________

Many thanks for completing this survey.

For more information about the study, you can visit our website [here](https://warwick.ac.uk/fac/sci/med/research/hscience/apc/qualityandsafety/gpnet-0/wp7/), or send the team an email at [GPNET0@warwick.ac.uk](mailto:GPNET0@warwick.ac.uk)

To receive updates about study progression, you can sign up to our mailing list by filling out this [form](https://warwick.us11.list-manage.com/subscribe?u=3551e6e82ec6e8f80c633958d&id=bc156f5f0c).
