# Supplementary figures and images for: Readiness to reduce primary care-associated carbon emissions in England: a cross-sectional survey of clinical and non-clinical staff views
Source: BMJ Open. 2025 Jul 18;15(7):e095457. doi: 10.1136/bmjopen-2024-095457 (PMC12273146; doi:10.1136/bmjopen-2024-095457)

**Supplemental file 3- Recruitment material**

**
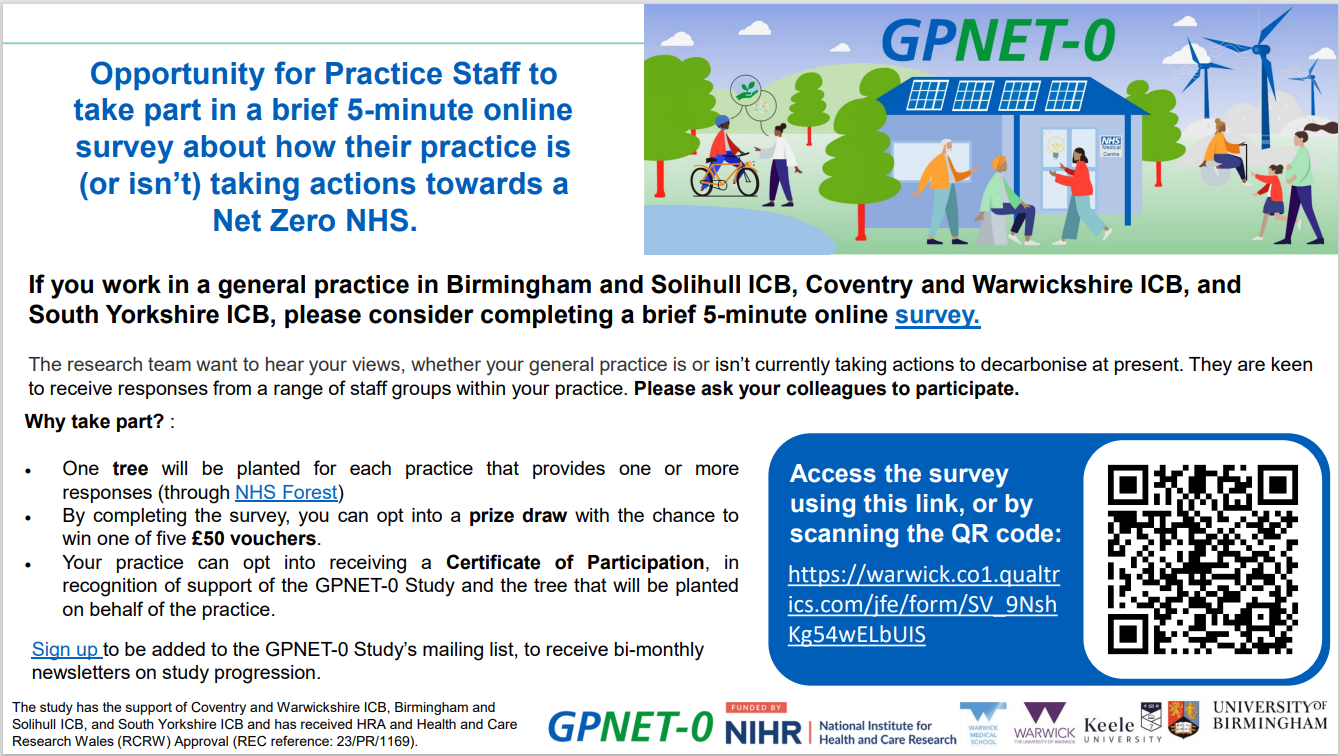
**

Supplement: online supplemental file 3 [file bmjopen-15-7-s003.docx]
