# Supplementary material for: Readiness to reduce primary care-associated carbon emissions in England: a cross-sectional survey of clinical and non-clinical staff views
Source: BMJ Open. 2025 Jul 18;15(7):e095457. doi: 10.1136/bmjopen-2024-095457 (PMC12273146; doi:10.1136/bmjopen-2024-095457)
Supplement: Supplementary file 4 [file bmjopen-2024-095457.reviewer_comments.pdf]

PEER REVIEW HISTORY

BMJ Open publishes all reviews undertaken for accepted manuscripts. Reviewers are asked to complete a checklist review form and are provided with free text boxes to elaborate on their assessment. These free text comments are reproduced below.

ARTICLE DETAILS

Title (Provisional)

Readiness to reduce primary care-associated carbon emissions in England: a cross-sectional survey of clinical and non-clinical staff views

Authors

Geddes, Olivia; Twohig, Helen; Dahlmann, Frederik; Eccles, Abigail; Karaba, Florence; Nunes, Ana Raquel; Spencer, Rachel; Dale, Jeremy

---

VERSION 1 - REVIEW

**Reviewer**	**1**
**Name**	**Simms, Nicole**
**Affiliation**	**University of Toronto, IHPME**
**Date**	**27-Dec-2024**
**COI**	**None**

This is a well-written (barring some minor typos that a close read will catch) paper that supports the assertions of similar studies examining the factors that contribute to the engagement of health sector workers in sustainability initiatives. The focus on general practice settings, multiprofessional staff, and uptake of sustainability-focused resources are welcome contributions. For consideration to further strengthen this work:

Analysis

1. My primary critique of the paper centres on the treatment of sustainability resources. Given the extent to which these are emphasized in the abstract and introduction, I was surprised at the relatively light touch they received in the body of the manuscript. The finding that resource uptake is higher when a sustainability lead is present is significant, but what is the extent to which these resources are actually used? Do practitioners find them helpful in initiating/sustaining/evaluating projects (in the absence of this information, I find your assertion in the "Implications for practice and policymakers" section that resources are important to achieving the NHS Net Zero target to be unsupported – though see my note #2)? Are you able to say anything about the types of resources that are most reported as used (toolkits, education modules, calculators, networks, etc.)? What are some of the other factors (beyond the presence of a sustainability lead) that might influence their uptake (such

as accessibility and relevance)? I realize some or even all of these questions may be deemed out of scope, and it seems unlikely that any further insights on resource use can be derived given the survey structure (though perhaps the free text responses could be instructive?), but I do think it would have add heft to the research findings if you are able to explore this a bit more fully (or at least point to the need to do so in future research).

Findings

2. The use of the term “resources” lacks clarity at times – at the outset, it refers to external resources that can be consulted to support sustainability efforts, but it is also used throughout to reference internal resources that are (or are not) devoted to sustainability work. Consider differentiating between these two items (perhaps “external” or “implementation” resources vs. “internal” resources?)

3. Related to the question of resource utility is the question of initiative efficacy. While I understanding the focus is on engagement vs impact, I think it may be prudent to note in your “Strengths and limitations” section that the presence of decarbonization initiatives does not necessarily equate to decarbonization, as not all of these will be successful or produce measurable impacts. I would have liked to see a question on the survey measuring participants' thoughts on the extent to which sustainability efforts are achieving their intended aims. If any of the free text responses speak to this, consider inclusion.

4. The relevance of the details provided in some of the statistical reporting is not immediately apparent. For example, you note that “107 (33.2%) respondents from 58 (35.6%) practices reported that they had a practice lead.” What is the significance of reporting the number of respondents (vs. simply stating that "respondents from x (%) practices reported that…") if you aren't going to enumerate instances in which respondents were unaware of a practice lead and/or incorrectly believed one was present.

References:

5. Consider updating Romanello reference (ref [1]): The 2024 report of the Lancet Countdown on health and climate change: facing record-breaking threats from delayed action. Romanello, Marina et al. The Lancet, Volume 404, Issue 10465, 1847 - 1896 https://www.thelancet.com/journals/lancet/article/PIIS0140-6736(24)01822-1/abstract

6. Regarding the text related to this resource in the “Background” section, consider using the term “food insecurity” instead of “food scarcity” as nutritional deficits can still emerge in the absence of scarcity if food is of insufficient quality or variety.

7. In citing international studies with similar conclusions on enabling/limiting factors in the “How this compares to other studies” section, consider adding the following (disclosure: I am an author on this paper): Luo, Owen & Razvi, Yasmeen & Kaur, Gurleen & Lim, MSc & Smith, Kelti & Carson, Jacob & Petrin-Desrosiers, Claudel & Haldane, Victoria & Simms, Nicole & Miller, Fiona. (2023). A qualitative study of what motivates and enables climate-

engaged physicians in Canada to engage in health-care sustainability, advocacy, and action. The Lancet Planetary Health. 7. e164-e171. 10.1016/S2542-5196(22)00311-4.

**Reviewer**	**2**
**Name**	**Rughwani, Hardik**
**Affiliation**	**Asian Institute of Gastroenterology, Medical Gastroenterology**
**Date**	**13-Mar-2025**
**COI**	**None**

I congratulate the authors of the article ‘Readiness to reduce general practice-associated carbon emissions in England: a cross-sectional survey of clinical and non-clinical staff views’ for the idea. The paper is well-conceptualized and well-written, but some issues must be addressed.

1. Please simplify the Results section of the Abstract. A lot of data is included, creating confusion.
2. In the Article Summary, kindly mention the complete form of ICB
3. In the Background section, please reference Global perspectives on Healthcare, carbon footprinting, and net zero targets of countries like the US, EU, China, and India.
4. The numbering of Ref 38 is not clear
5. The Study aims should be mentioned under the Methods section
6. Ref 42 is not correctly mentioned. “see Sustainability is not clear”
7. In the Data analysis section, taking in multiple responses has added bias to the study. Kindly address this issue by statistical analysis (multivariate analysis or uncertainty index) to bring in uniformity of data. If unable to do so, kindly mention the limitations in the Discussion section. As such, the system should have been created so that multiple responses would not have been recorded.
8. Some tables are not required. It can be removed or placed as supplementary materials, as shown in Table 5.
9. Showing the importance of Leadership and Incentives is a valid and valuable point. Recommendations like the establishment of a ‘Sustainability department’ and leadership under ‘Eco-Visionaries’ can be recommended. Recent GUT-BMJ articles can be quoted.

Also, in the suggestions section, please mention that further studies should be undertaken with Practitioners of various subspecialties like cardiology, gastroenterology, and surgery, as these perspectives about environment and health are lacking overall. So, it needs awareness at all levels.

VERSION 1 - AUTHOR RESPONSE

Reviewer: 1<br>This is a well-written (barring some minor typos that a close read will catch) paper that supports the assertions of similar studies examining the factors that contribute to the engagement of health sector workers in sustainability initiatives. The focus on general practice settings, multiprofessional staff, and uptake of sustainability-focused resources are welcome contributions.	Thank you for these positive comments. We have carefully proof read the paper to correct minor typos.
1. My primary critique of the paper centres on the treatment of sustainability resources. Given the extent to which these are emphasized in the abstract and introduction, I was surprised at the relatively light touch they received in the body of the manuscript. The finding that resource uptake is higher when a sustainability lead is present is significant, but what is the extent to which these resources are actually used? Do practitioners find them helpful in initiating/sustaining/evaluating projects (in the absence of this information, I find your assertion in the "Implications for practice and policymakers" section that resources are important to achieving the NHS Net Zero target to be unsupported – though see my note #2)? Are you able to say anything about the types of resources that are most reported as used (toolkits, education modules, calculators, networks, etc.)? What are some of the other factors (beyond the presence of a sustainability lead) that might influence their uptake (such as accessibility and relevance)? I realize some or even all of these questions may be deemed out of scope, and it seems unlikely that any further insights on resource use can be derived given the survey structure (though perhaps the free text responses could be instructive?), but I do think it would have add heft to the research findings if you are able to explore this a bit more fully (or at least point to the need to do so in future research).	Thank you for highlighting these points which we have carefully considered. We agree about their importance, but that many go beyond the scope of this study. We have made several small changes to the text in light of them, including making more explicit the scope of the research that we are currently undertaking, and how this is designed to address many of these questions.<br>We have added the following to the Discussion<br>Finally, we were unable to assess the acceptability and extent of the activities reported as having occurred within practices, how resource materials had been used and, most importantly, the measurable impact that this had. It cannot be assumed that all initiated activities were undertaken successfully in terms of achieving their intended purpose. While this was beyond the scope of this survey, this is currently being explored through longitudinal investigation as part of the GPNET-0 study [45].

	We have also added some detail about the GPNET-0 study to the end of the conclusion:<br>This study is undertaking an in-depth exploration that builds on the findings of this survey to address key questions relating to the system-wide factors that influence the uptake and implementation of decarbonisation activities in general practice.
2. The use of the term "resources" lacks clarity at times – at the outset, it refers to external resources that can be consulted to support sustainability efforts, but it is also used throughout to reference internal resources that are (or are not) devoted to sustainability work . Consider differentiating between these two items (perhaps "external" or "implementation" resources vs. "internal" resources?)	We have made changes to the text to make this distinction clearer.
3. Related to the question of resource utility is the question of initiative efficacy. While I understanding the focus is on engagement vs impact, I think it may be prudent to note in your "Strengths and limitations" section that the presence of decarbonization initiatives does not necessarily equate to decarbonization, as not all of these will be successful or produce measurable impacts. I would have liked to see a question on the survey measuring participants' thoughts on the extent to which sustainability efforts are achieving their intended aims. If any of the free text responses speak to this, consider inclusion.	We agree with the importance of this consideration and have included this within the changes made to the strengths and limitations highlighted above.
4. The relevance of the details provided in some of the statistical reporting is not immediately apparent. For example, you note that "107 (33.2%) respondents from 58 (35.6%) practices reported that they had a practice lead." What is the significance of reporting the number of respondents (vs. simply stating that "respondents from x (%) practices reported that...") if you aren't going to enumerate instances in which respondents were unaware of a practice lead and/or incorrectly believed one was present.	We have revised some of the details in the statistical reporting in light of this comment.
5. Consider updating Romanello reference (ref [1]): The 2024 report of the Lancet Countdown on health and climate change: facing record-breaking threats from delayed action. Romanello, Marina et al. The Lancet, Volume 404, Issue 10465, 1847 - 1896	Thank you for this suggestion. We have revised the reference accordingly

6. Regarding the text related to this resource in the "Background" section, consider using the term "food insecurity" instead of "food scarcity" as nutritional deficits can still emerge in the absence of scarcity if food is of insufficient quality or variety.	Thank you. We have made this change.
7. In citing international studies with similar conclusions on enabling/limiting factors in the "How this compares to other studies" section, consider adding the following (disclosure: I am an author on this paper): Luo, Owen & Razvi, Yasmeen & Kaur, Gurleen & Lim, MSc & Smith, Kelti & Carson, Jacob & Petrin-Desrosiers, Claudel & Haldane, Victoria & Simms, Nicole & Miller, Fiona. (2023). A qualitative study of what motivates and enables climate-engaged physicians in Canada to engage in health-care sustainability, advocacy, and action. The Lancet Planetary Health. 7. e164-e171. 10.1016/S2542-5196(22)00311-4.	We have made an addition to the end of this section of the Discussion and referred to this paper:<br>Such factors not only affect general practice, but have also been shown to apply across other healthcare settings (Luo et al)
Reviewer: 2<br>Dr. Hardik Rughwani, Asian Institute of Gastroenterology<br>Comments to the Author:<br>I congratulate the authors of the article 'Readiness to reduce general practice-associated carbon emissions in England: a cross-sectional survey of clinical and non-clinical staff views' for the idea. The paper is well-conceptualized and well-written, but some issues must be addressed.	Many thanks for this positive comment.
1. Please simplify the Results section of the Abstract. A lot of data is included, creating confusion.	We have attempted to simplify this and hope it is now clearer.
2. In the Article Summary, kindly mention the complete form of ICB	Done
3. In the Background section, please reference Global perspectives on Healthcare, carbon footprinting, and net zero targets of countries like the US, EU, China, and India.	We feel there is a risk of the Background section becoming over inclusive and lengthy, but have added a sentence to give some wider context
4. The numbering of Ref 38 is not clear	Corrected
5. The Study aims should be mentioned under the Methods section	We feel that they better fit with the background, but would be happy to make a change if the Editor felt they would be best placed in the methods
6. Ref 42 is not correctly mentioned. "see Sustainability is not clear"	Corrected
7. In the Data analysis section, taking in multiple responses has added bias to the study. Kindly address this issue by statistical analysis (multivariate analysis or uncertainty index) to bring in uniformity of data. If unable to do so, kindly mention the limitations in the Discussion section. As such,	We have edited the strengths and limitations section to explain our approach and its limitations more clearly:

the system should have been created so that multiple responses would not have been recorded.	A strength was that we did not limit the number of staff members from each practice that could participate in the survey as wanted to capture diverse perspectives on decarbonisation plans and activities. This approach allowed us to see that, within practices that had several respondents, there were significant differences in opinions and varying levels of awareness about their approach to decarbonisation. To generate a single practice-level response for questions about specific decarbonisation actions, either taken or planned, we assumed that if at least one respondent from that practice reported a positive answer, then this was a correct indicator, in contrast to any negative responses from other staff members at the same practice which were attributed to lack of awareness. While this pragmatic, amalgamative approach helped address inconsistent internal knowledge, it may have led to some inaccuracies. This is especially likely in practices with only one respondent, particularly if that individual did not hold a leadership or managerial role related to decarbonisation and was therefore less aware about initiatives led by others.

8. Some tables are not required. It can be removed or placed as supplementary materials, as shown in Table 5.	We are uncertain about which tables are felt to be unnecessary. We are happy to be guided by the editor about which should be removed or placed as supplementary materials
9. Showing the importance of Leadership and Incentives is a valid and valuable point. Recommendations like the establishment of a 'Sustainability department' and leadership under 'Eco-Visionaries' can be recommended. Recent GUT-BMJ articles can be quoted.	We recognise the relevance of this comment and have revised the first two sentences of the section 'Implications for practice and policymakers' accordingly.
10. Also, in the suggestions section, please mention that further studies should be undertaken with Practitioners of various subspecialties like cardiology, gastroenterology, and surgery, as these perspectives about environment and health are lacking overall. So, it needs awareness at all levels.	While we agree with the need for similar studies to be undertaken across other specialties, we have not added this to our recommendations as we do not feel that this can be derived from our findings.

VERSION 2 - REVIEW

Reviewer 2

Name Rughwani, Hardik

Affiliation Asian Institute of Gastroenterology, Medical Gastroenterology

Date 13-Jun-2025

COI

Can be accepted as changes done as suggested
